# The Rest Cure

**Published:** 1901-10-05

**Authors:** 


### THE REST CURE.

Lecturing recently at the London Polytechnic, Dr. W. S. Playfair pointed out the disastrous consequences of those forms of chronic invalidism in women which we associate with the terms hysteria, nervousness, hypochondriasis, and neurasthenia. These nervous states are mainly found in the cultured and educated classes, and they are apt to be developed by injudicious education, especially in those who inherit a mobile and ill-balanced nervous constitution. Numerous as are the causes of neurasthenia it will generally be found that defective nutrition on the one side, and on the other some form of mental or emotional disturbance play an important part in the production of this disorder, and in the treatment of such cases one must aim at the restoration of health to the body as well as at restoring the balance of the nervous system. The method of treatment originated by Weir-Mitchell is the most generally successful way of dealing with these cases. But it needs to be carefully and thoroughly applied. A frequent cause of failure is the attempt to carry out the treatment "in a modified way" so as to meet objections raised by the patient or her friends, thus omitting some essential part. It is, for example, a *sine quâ non* that the patient shall be removed from her home surroundings, yet often an attempt is made to practise the treatment at home and to allow the occasional visits of relatives and friends. Almost all such attempts end in failure. The rest cure, then, has two aims, the treatment of the bodily and of the mental conditions. In most neurasthenic patients the general nutrition is in a wretched state. It is no use *telling* the patient to take food. Something must be done to secure nutrition independently of the will of the patient. That something is massage, which produces nutritive changes in the muscles and thus enables the patient to take food. The test of massage being well done is not the employment of high-sounding and misleading names, which only serve to obscure a very simple thing, but the bestowal on the patient of the capacity to take large quantities of food. The psychological aspect of the treatment is as important as the physical, and here comes in the necessity of removing the patient from the influence of well meaning but injudicious friends, and of excluding her from all communication with her relatives, at least for a time. After six weeks or so in bed the patient may get up for a few hours daily, and attended by the nurse, should walk out and begin to attend church, concerts, theatres, etc., and when possible it is very desirable that before returning to her former surroundings she should go away with a proper attendant on a sea voyage or to the seaside or the country. This treatment is expensive, but it is less expensive than prolonged and constant ill-health.

